# A Recombinant HAV Expressing a Neutralization Epitope of HEV Induces Immune Response against HAV and HEV in Mice

**DOI:** 10.3390/v9090260

**Published:** 2017-09-15

**Authors:** Xiang Kui, Kusov Yuri, Ying Guan, Yan Wang, Shan Yi, Jinyuan Wu, Na Yin, Yan Zhou, Hongjun Li, Maosheng Sun

**Affiliations:** 1 Department of Molecular Biology, Institute of Medical Biology, Chinese Academy of Medical Sciences, 935 Jiaoling Road, Kunming 650118, China; kuixiang@foxmail.com (X.K.); yi_shan@126.com (S.Y.); wujinyuan@imbcams.com.cn (J.W.); yinna@imbcams.com.cn (N.Y.); yanyan_850@163.com (Y.Z.); 2 Department of Pathology, The Second Affiliated Hospital of Kunming Medical University, 374 Dianmian Road, Kunming 650101, China; wyan_sky@163.com; 3 Institute of Biochemistry, Center for Structural and Cell Biology in Medicine, University of Lübeck, Ratzeburger Allee 160, 23562 Lübeck, Germany; koussov@vuz.uni-luebeck.de; 4 German Center for Infection Research (DZIF), Hamburg-Lübeck-Borstel-Riems Site, University of Lübeck, 23562 Lübeck, Germany; 5 China Tobacco Yunnan Industrial Co., Ltd., 41 Keyi Road, Kunming 650106, China; guan_ying1984@163.com

**Keywords:** combined vaccine, hepatitis A, hepatitis E, virus, vector, neutralization epitope

## Abstract

Hepatitis A virus (HAV) and hepatitis E virus (HEV) are causative agents of acute viral hepatitis transmitted via the fecal–oral route. Both viruses place a heavy burden on the public health and economy of developing countries. To test the possibility that HAV could be used as an expression vector for the development of a combination vaccine against hepatitis A and E infections, recombinant HAV-HEp148 was created as a vector to express an HEV neutralization epitope (HEp148) located at aa 459–606 of the HEV capsid protein. The recombinant virus expressed the HEp148 protein in a partially dimerized state in HAV-susceptible cells. Immunization with the HAV-HEp148 virus induced a strong HAV- and HEV-specific immune response in mice. Thus, the present study demonstrates a novel approach to the development of a combined hepatitis A and E vaccine.

## 1. Introduction

Hepatitis A virus (HAV) and hepatitis E virus (HEV) are causative agents of acute viral hepatitis. HAV is a small, non-enveloped virus with a positive-sense RNA in the family *Picornaviridae* [1]. HAV infection is responsible for approximately 1.4 million new cases worldwide each year. Serologic evidence suggests that tens of millions of new HAV infections occur each year [2]. HEV is a non-enveloped positive-sense virus in the *Orthohepevirus* (Species A) genus of the *Hepeviridae* family, according to the new nomenclature [3]. A study of global disease burdens estimated that HEV accounts for approximately 20.1 million HEV infections, 70,000 deaths, and 3000 stillbirths, annually [4]. In Western China, HAV transmission predominately occurs among children, whereas in Eastern, HAV infects adults with higher infection rates [5]. Approximately 20% of HEV-infected pregnant women die, due to liver failure [6,7]. Both HAV and HEV are enterically transmitted viruses, and have similar clinical symptoms and epidemiological features. It is not possible to distinguish between these viruses using clinical or biochemical parameters. Co-infection with HAV and HEV, or simultaneous HAV and HEV outbreaks due to contaminated water supplies, have been reported [8,9]. 

Hepatitis A vaccines (HA vaccines) have been commercially available since the 1990s. In 1979, Provost and Hilleman propagated HAV via cell culture, which laid the foundation for the development of the inactivated and live attenuated HA vaccines [10]. Both the inactivated and live attenuated HA vaccines are highly effective in preventing hepatitis A infection [11,12,13]. The attenuated live vaccine was capable of providing protection during a hepatitis A outbreak, and for up to 15 years [12,14,15]. The first licensed recombinant hepatitis E vaccine (HE vaccine) was derived from the immunogenic region of amino acid residues (aa) 452–617 of the HEV capsid protein, which is encoded by open reading frame 2 (ORF2) [16,17]. Most conformation-dependent monoclonal antibodies (McAbs) recognize the surface epitopes of native HEV, which are located at aa 459–606 of the HEV capsid protein [18]. Further research showed that the hydrophobic region at position aa 597–602 is intimately involved in the formation of important conformational neutralizing epitopes [19,20]. These homodimer epitopes did not react with neutralizing McAbs after denaturation into monomers [21]. Therefore, dimerization is important for an effective presentation of HEV neutralization epitopes. 

Combined vaccines against two or more diseases reduce immunization programs and the cost of vaccination, and improve compliance. Some studies have tested the possibility of developing a combined vaccine against hepatitis A and E. Dong et al. demonstrated that a combined HA and HE vaccine induced anti-HAV and HEV neutralizing antibodies effectively [22]. Furthermore, the inactivated HA vaccine improved the immune efficiency of the recombinant HE vaccine, in the combined HA and HE vaccine [22]. Similar results have also been reported in studies investigating recombinant subunit vaccines based on HAV and HEV epitopes [23,24]. 

Previously, a recombinant HAV was tested as a vector for the expression of foreign proteins. HAV constructs presenting a human immunodeficiency virus gp41 epitope induced a gp41-specific immune response [25]. Viable HAV particles can package full-length viral genomes that contain insertions of approximately 600 nucleotides (nts) at the 2A/2B junction and maintain infectivity [26]. Therefore, HAV could be used as an expression vector for the development of cost-effective combination vaccines. In this study, we verified this hypothesis by first creating a recombinant chimeric HAV (HAV-HEp148) expressing the HEV neutralization epitope located at aa 459–606 of the HEV capsid protein (HEp148). The data show that the HAV-HEp148 vector could induce similar antibody responses against HAV and HEV in immunized mice, compared with the antibody response observed after monovalent vaccination. Our results demonstrate a novel approach to the development of combined vaccines against different diseases.

## 2. Materials and Methods

### 2.1. Cell Cultures

*Escherichia coli* strain DH5α was maintained in our laboratory. All cell lines were grown in growth medium supplemented with 10% fetal bovine serum. Human hepatoma Huh-7 cells, obtained from OBIO Technology Co. Ltd. (Shanghai, China), were grown in Dulbecco’s modified Eagle’s medium (DMEM). The Vero cell line (derived from African green monkey kidneys) was maintained in our laboratory and was grown in Eagle’s minimal essential medium (MEM). 

### 2.2. Plasmids and Constructs

The plasmid pT7-HAV-A60 expressing the full-length HAV genome (strain 18f, GenBank accession number M59808) with elongated poly-A tail under control of the T7 promoter was described previously [27]. The plasmid pEHG2 (containing HEV ORF2) was maintained in our laboratory.

### 2.3. pT7-HAV-Multiple Cloning Site 

**Figure 1:**
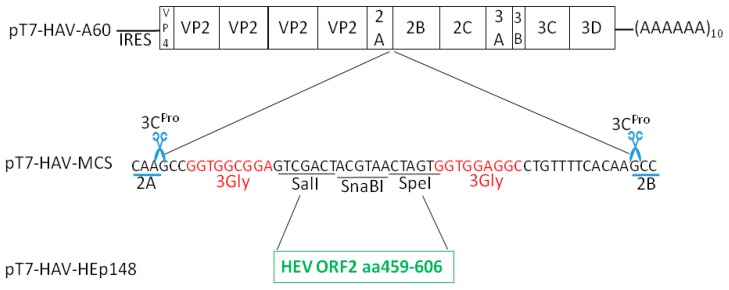
Schematic representation of hepatitis A virus-HEV neutralization epitope (HAV-HEp148) constructs containing the HEp148 protein in the 2A/2B junction of HAV. The HEp148 gene was cloned into the 2A/2B junction of the HAV genome in the pT7-HAV-A60-MCS (multiple cloning site). This plasmid contains a polylinker coding for restriction sites (SalI, SnaBI, and SpeI) flanked by 3Gly hinges (red color letters), and HAV 3Cpro cleavage sites (scissors) engineered into the 2A/2B junction of pT7-HAV-A60. The HEp148 gene coding for the hepatitis E virus (HEV) neutralization epitope was cloned into the SalI and SpeI sites of pT7-HAV-A60-MCS, and the resulting plasmid was termed pT7-HAV-HEp148.

The plasmid pT7-HAV-A60 was modified by inserting unique restriction sites for SalI, SnaBI, and SpeI at the 2A/2B junction, previously proposed to accommodate exogenous sequences in the HAV genome [28]. The restriction sites are flanked on each side by nucleotide sequences encoding consensus 3Cpro cleavage sites. In order to increase polypeptide chain flexibility and enhance 3Cpro cleavage, nucleotide sequences coding for three-residue Gly (3-Gly) hinges were introduced between the restriction sites and 3Cpro cleavage sites [27,28]. To construct this plasmid, a synthetic polylinker containing the three restriction sites flanked by nucleotides coding for 3-Gly linkers and 3Cpro cleavage sites, was cloned into the 2A/2B junction of pT7-HAV-A60 using recombination-based cloning technologies (In-Fusion HD Cloning Kit, Clontech, Mountain View, CA, USA) [29] (Figure 1). 

### 2.4. pT7-HAV-HEp148

The HEV epitope gene of 444 bp coding for the HEp148 protein from the pEHG2 plasmid was cloned into the multiple cloning site of the pT7-HAV-MCS (multiple cloning site) polylinker as described above. A DNA fragment was amplified from pEHG2 using the synthetic primers 5′-CGGTGGCGGAGTCGACTCGCGCCCCTTTTCTGTCCTCCG-3′ and 5′-GGCCTCCACCACTAGTCGCAGAGTGGGGGGCTAAAACAGCAAC-3′, which introduced SalI and SpeI restriction sites at the 5′ and 3′ ends of the gene, and 15 bases of homology to the recombination site of pT7-HAV-MCS, respectively. The PCR fragment was gel-purified and cloned into pT7-HAV-MCS linearized with SalI and SpeI using the In-Fusion method [29]. The resulting construct, coding for the HEV epitope of the protein HEp148 inserted into the 2A/2B junction in frame with the rest of the HAV polyprotein, was termed pT7-HAV-HEp148 (Figure 1).

### 2.5. RNA Transcription and Transfection

The replicon plasmid pT7-HAV-HEp148 was cut at the unique AgeI site located at the 3′ end of the HAV sequence. The T7 MEGAscript^®^ in vitro transcription kit (Thermo Fisher Scientific, Waltham, MA, USA) was used to transcribe viral RNA from the linearized pT7-HAV-HEp148, according to the manufacturer’s suggested protocol. Huh-7 cells were transfected with in vitro-synthesized RNA transcripts containing an elongated poly(A) tail (A60) using a liposome-mediated transfection procedure (Lipofectamine 3000, Thermo Fisher Scientific). Briefly, 7 µg of RNA was added to 17 µL of Lipofectamine 3000 in a total volume of 500 µL adjusted with Opti-MEM (Gibco^®^, Thermo Fisher Scientific), and the mixture was incubated for 5 min, after which the RNA–Lipofectamine mixture was added dropwise to nearly confluent monolayers of Huh-7 cells in 60 mm diameter dishes. Following 5 to 6 h of incubation, the transfection mixture was removed from the cells and the cells were washed twice with phosphate-buffered saline (PBS) and fed with normal growth medium. Twelve to 14 days following transfection, transfected Huh-7 cells were mechanically scraped into 3 mL of PBS and subjected to three freeze–thaw cycles. Nuclei and cell debris were removed by centrifugation at 1000× *g*. Supernatant was extracted with an equal volume of chloroform and filtered through a 0.22-µm filter. The harvested virus was named HAV-HEp148 and stored at −80 °C until use.

### 2.6. RT-PCR and Sequence Analysis of Viral RNA

Viral RNA was isolated from harvested virus using the Mini BEST Viral RNA/DNA Extraction Kit Ver. 5.0 (Takara, Takara Biomedical Technology, Beijing, China) and suspended in DNase/RNase-free water. Reverse transcription (RT)-PCR was carried out using the HiScript II One Step RT-PCR Kit (Vazyme, Vazyme Biotech Co., Ltd., Nanjing, China) according to the manufacturer’s recommendations. The forward and reverse primers coded for HAV nts 3137 to 3161, and 3544 to 3568, which corresponded to 432 bp of the 2A/2B junction. In case of the successful cloning of the HEp148 gene into the 2A/2B junction, the size of RT-PCR fragment should correspond to 876 bps. The amplified DNA fragment was gel-purified and sequenced by Sangon Biotech Corporation (Shanghai, China).

### 2.7. Immunofluorescence for HAV and HEV Capsid Protein

Huh-7 cells infected with the HAV-HEp148 virus were maintained at 35 °C for 7 days before processing. Mock and transfected cells grown on glass coverslips in a 12-well format were fixed with cold acetone for 30 min, air dried, blocked with 2% bovine serum albumin in PBS, and stained with HAV-vaccinated rabbit antiserum (produced in our laboratory) and anti-HEV ORF2 mouse monoclonal antibodies (Merck Millipore Corporation, Darmstadt, Germany). At the same time, negative rabbit and mouse serum (produced in our laboratory) were used as negative controls for antibodies in transfected cells. Following three washes with PBS, cells were stained with fluorescein isothiocyanate (FITC)-conjugated goat anti-rabbit (HAV) and DyLight 549-conjugated goat anti-mouse (HEV capsid protein) secondary antibodies (Kirkegaard & Perry Laboratories, Gaithersburg, MD, USA). The cells were washed again and stained with 4′,6-diamidino-2-phenylindole (DAPI). Fluorescent micrographs were taken with a Nikon microscope at 400× magnification and processed with NIS ELEMENTS (version 4.30.01, Nikon) and Adobe Photoshop 6.0 software.

### 2.8. Non-Denaturing Gel Electrophoresis and Western Blotting

Cytoplasmic extracts were prepared at 12–14 days post-infection from cells infected with HAV-HEp148 or mock-infected by lysis in 200 µL of RIPA lysis buffer (Beyotime Biotechnology, Jiangsu, China). Briefly, 20 µL aliquots, with or without heat treatment, were separated by non-denaturing-12% polyacrylamide gel electrophoresis (12% PAGE) and then transferred to nitrocellulose membranes. After blocking by incubation in PBS containing 0.1% Tween 20 (PBST) and 5% skim milk for 2 h at room temperature, the membranes were probed overnight at 4 °C with anti-HEV capsid rabbit polyclonal antibodies (WNT Biological Co., Ltd., Kunming, China, 1:200 dilution) and mouse monoclonal antibody to β-actin (Abgent Corporation, San Diego, CA, USA, 1:1000 dilution). Signals from the membranes were detected using HRP-conjugated goat anti-rabbit (HEV capsid) and goat anti-mouse (β-actin) IgG (Merck Millipore, 1:2000 dilution) and enhanced chemiluminescence (Merck Millipore).

### 2.9. Recombinant HAV Amplification and Titer Determination

The purified HAV-HEp148 virus was diluted 1:100 in MEM to infect confluent monolayers of Vero cells in 175 cm^3^ flasks for 6 h at 37 °C. Maintenance medium with 2% newborn calf serum was added, and infected cells were incubated at 35 °C in a CO_2_ incubator for 3 weeks. The virus was harvested on day 21 after infection, and then extracted with chloroform and filtered through a 0.22 µm filter. HAV titers were determined by inoculating the confluent monolayer of cells cultured in 25 cm^3^ culture flasks, and infection was verified by a double-antibody sandwich enzyme-linked immunosorbent assay (ELISA). Briefly, four replicate flasks were inoculated with 1 mL of 10-fold dilutions of HAV for 2 h and cultured in MEM with 2% newborn calf serum for 3 weeks at 35 °C. After virus harvest, a commercial ELISA kit (qualitative manual method; WNT Biological Co., Ltd., Kunming, China) was used to determine the 50% cell culture infectious titers (CCID_50_/mL) according to the method previously described by Reed and Muench [30,31,32]. The infectious titer of the HAV-HEp148 viruses was found to be 7.5 log CCID_50_/mL as determined by the ELISA.

### 2.10. Immunogenicity Analysis of the Recombinant Virus and Serum Collection

The protocol for use of animals was approved by the Ethics Committee of the Institute of Medical Biology, and all the procedures were carried out in accordance with the approved guidelines (with code VDWSP2017035). Five- to 6-week old female Balb/c mice were obtained from the Institute of Medical Biology, Chinese Academy of Medical Sciences (Kunming, China). All mice were maintained in the animal facilities of the Institute of Medical Biology and cared in accordance with the guidelines of the Institutional Animal Care and Use Committee. Twenty-four mice were randomly assigned to four vaccination groups as follows: (1) 100 µL of 6.5 logCCID_50_/mL HAV-HEp148 virus; (2) 100 µL of 6.5 logCCID_50_/mL live attenuated HA vaccine (provided by the Institute of Medical Biology; Chinese Academy of Medical Sciences, Kunming, China); (3) 100 µL of 30 µg/mL recombinant HE vaccine (purchased from the Yunnan Center for Disease Control and Prevention); and (4) 100 µL of PBS. Each mouse was injected subcutaneously and then boosted with the same dose 4 weeks later. Pre-immune serum samples were collected before immunization. Post-immune sera were obtained at weeks 2, 4, 6, and 8 after the first immunization. All sera were stored at −80 °C until testing.

### 2.11. Anti-HAV and HEV Antibody Detection

Serum IgG antibody responses against HAV and HEV were monitored by using a commercial indirect ELISA kit (WNT Biological Co., Ltd., Kunming, China) according to the manufacturer’s protocol. Serum samples were added in 2-fold serial dilutions starting from 1:20. The positive cut-off value was 2.1-times OD450 value over the normal negative controls. The antibody titers were determined by the highest dilution of samples showing a 2.1 times OD450 value over controls. The effective antibody dose was determined by the maximum positive dilution and reported as geometric mean titer (GMT). ELISA data were processed and graphed using GraphPad Prism 6 software. Statistical significance was determined using the Holm–Sidak multiple *t*-test method. *p* Values of <0.05 were considered to be statistically significant.

## 3. Results

### 3.1. Insertions at the 2A/2B Junction

A 51 nt polylinker, containing a 3Gly–MCS (SalI, SnaBI, and SpeI)–3Gly and consensus 3Cpro cleavage sites, was constructed at the 2A/2B junction of pT7-HAV. A 444 nts fragment coding for the HEp148 protein was then inserted into the polylinker of pT7-HAV-MCS, and the resulting construct was termed pT7-HAV-HEp148 (Figure 1). The sequencing results showed that the 444 nts sequence was inserted into the polylinker at the 2A/2B junction of pT7-HAV, as expected, and that the reading frame was correct.

### 3.2. Rescue of Recombinant Hepatitis A Virus

**Figure 2:**
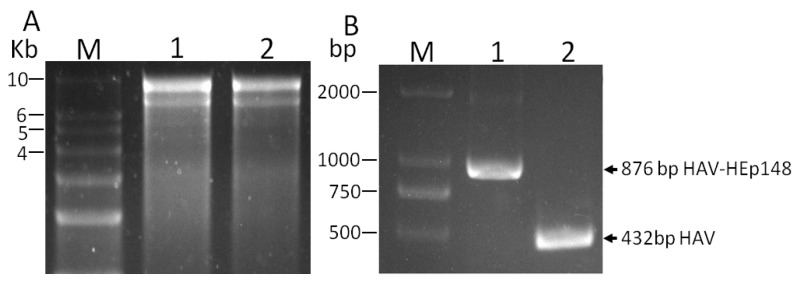
Rescue of the HAV constructs containing the HEp148 gene at the 2A/2B junction. (**A**) RNA transcribed from pT7-HAV-HEp148 with T7 RNA polymerase. Lane M indicates RNA size markers, lane 1 indicates plus-strand RNA of the HAV-HEp148 virus, and lane 2 indicates plus-strand RNA from the wild-type HAV virus. The sizes of the marker bands are in kb; (**B**) RT-PCR results show the HEp148 insertion. Viral RNA was extracted and fragments were amplified with RT-PCR using HAV primers specific for HAV nucleotides (nts) 3137 to 3161 and 3544 to 3568, spanning the HEp148 insertion. RT-PCR fragments amplified from RNA extracted from HAV-HEp148 (lane 1) and wild type HAV (lane 2) were analyzed with 1.5% agarose gel electrophoresis. The RT-PCR fragments from HAV-HEp148 and parental HAV are indicated by arrowheads, and their sizes given in base pairs (bp). The size of the DNA molecular weight markers (lane M) is indicated in thousand base pairs (kb).

**Figure 3:**
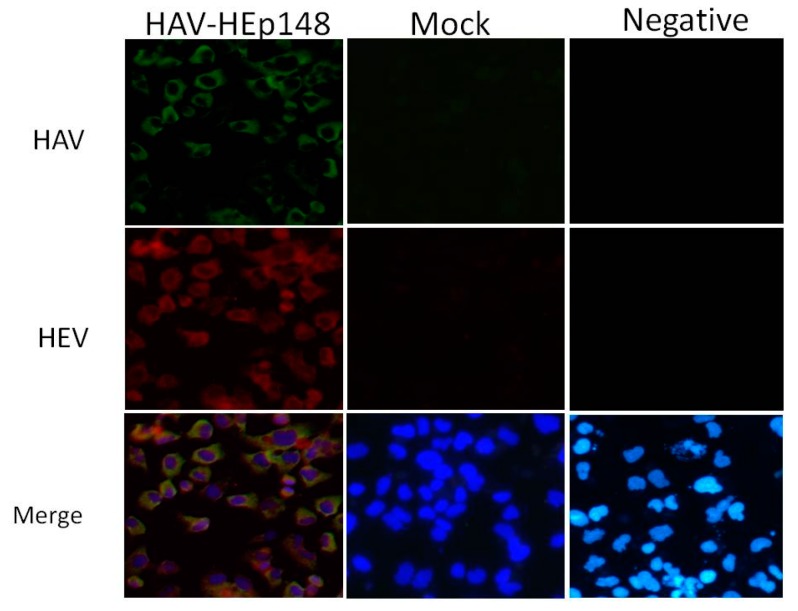
Immunofluorescence (IF) analysis of HAV-HEp148-infected Huh-7 cells. Monolayers of Huh-7 cells were either infected with HAV-HEp148 or mock-infected, fixed with acetone at 7 days post infection (p.i.), and stained with antiserum directed against HAV (green), and antibodies against HEV capsid (ORF2) protein (red). Negative serum was used as a negative control for antibodies in transfected cells. Merged images also show nuclear labeling with DAPI. Micrographs were taken with a Nikon microscope at a 400× magnification.

To rescue viruses, T7 RNA polymerase was used to transcribe the HAV RNA, in vitro, from linearized pT7-HAV-HEp148. The predominant RNA species from the transcription reaction was 7.5 kb in length, but an additional band, attributed to premature transcription termination, was also noted (Figure 2A). A similar result was observed when HAV and poliovirus cDNA were transcribed with SP6 or T7 polymerases [33,34]. At 12 to 14 days following transfection of the HAV construct transcripts, recombinant HAV-HEp148 virus was harvested and assayed using RT-PCR and immunofluorescence (IF). The RT-PCR results show that the rescued virus carried the HEp148 gene (Figure 2B). Further sequencing results showed that this fragment was inserted in line with expectations, did not carry mutations, and was in the correct reading frame. IF analysis showed that HAV-HEp148 could infect Vero cells, and the HEV antigen HEp148 was expressed in these cells (Figure 3).

### 3.3. Western Blot Identification of the HEV ORF2 Antigen

**Figure 4:**
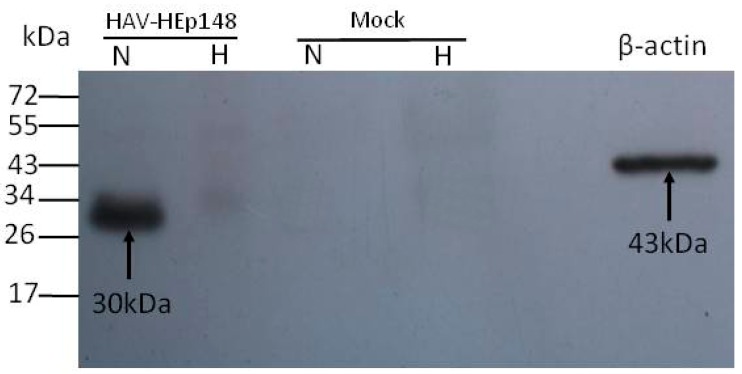
Western blot analysis of the HEp148 antigen expressed by HAV-HEp148 virus in Huh-7 cells. The cell extracts were separated by 12% PAGE gel, proteins were transferred onto nitrocellulose membranes and probed with anti-HEV capsid rabbit polyclonal antibodies. HAV-HEp148: HAV-HEp148 infected samples. Mock: Mock-infected samples. N: samples were not heated. H: samples were boiled for 10 min. Molecular mass markers are shown in kDa on the left margin. HEp148 protein dimer (30 kDa) andβ-actin (43 kDa) are indicated by arrowheads.

Western blot analysis was used to confirm the immunoreactivity of HEV capsid antigen expressed by HAV-HEp148 viruses after infection of Huh-7 cells. Mock-infected Huh-7 cells were used as a negative control. The HAV-HEp148-infected sample without heat treatment indicates the presence of dimers corresponding to a 30 kDa band. However, there is no reactive band at 15 kDa, corresponding to monomers, in samples with and without heat treatment (Figure 4).

### 3.4. Recombinant Virus Elicits HAV- and HEV-Specific IgG

**Figure 5:**
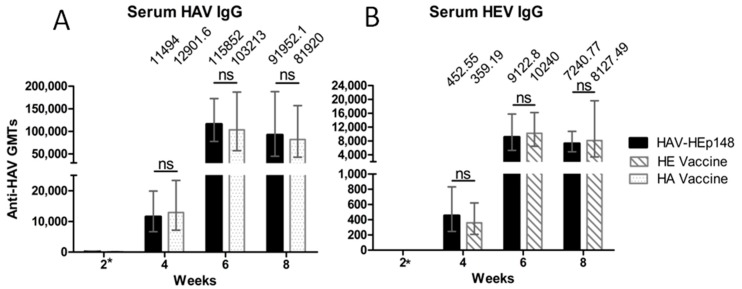
Profiles of HAV- and HEV-specific IgG production in humoral immune responses following vaccination. (**A**) Anti-HAV IgG and (**B**) anti-HEV IgG were detected by indirect ELISA after immunization of the immunized groups, as indicated. Each serum effective dose is the reciprocal value of the maximum positive dilution. The geometric mean titer (GMT) values are presented at the tops of the graphs. Error bars represent the 95% confidence level (Cl). *, IgG cannot be detected at 1:80 serum dilution. ns, no significant difference.

To evaluate whether the HAV-HEp148 virus could induce IgG responses against both HAV and HEV in mice, blood samples were collected at weeks 2, 4, 6, and 8, after the first immunization. Serum anti-HAV and HEV-specific IgG antibodies were determined by an ELISA assay using a commercial indirect ELISA kit (WNT Biological Co., Ltd., Kunming, China). The antibody GMT values, plotted against the immunization period, are shown in Figure 5. The HAV-HEp148, HA vaccine and HE vaccine groups displayed apparent immune responses beginning at week 4. After the second immunization, the HAV IgG titer in the HAV-HEp148 and HA vaccine groups increased to 115,852 and 103,213, respectively (Figure 5A). No statistically significant difference in titers of HAV-specific IgG was noted between the HAV-HEp148 and HA vaccine groups from weeks 4 to 8. As shown in the data presented in Figure 5B, the HEV IgG titers of the HAV-HEp148 and HE vaccine groups increased to 9122.8 and 10,240, respectively. There was also no significant difference in the HEV IgG titers between the HAV-HEp148 and HE vaccine groups. The PBS group did not generate significant antibody responses against either the HAV or HEV antigens. These results indicate that the HAV-HEp148 group could elicit similar humoral immune responses, as those observed in the HA or HE vaccine groups. 

## 4. Discussion

Combined vaccination against HAV and HEV was considered desirable, because both HAV and HEV are transmitted by the fecal–oral route, and share many epidemiological and clinical features. Classical combined vaccines are composed of two or more separate vaccines against different pathogens, and it is necessary to investigate the compatibility of different vaccines. In this study, we constructed a novel combination vaccine against HAV and HEV using an HAV vector. RT-PCR and IF analyses showed that the recombinant HAV, HAV-HEp148, could proliferate in HAV-susceptible cells and express the HEp148 polypeptide. 

During HEV assembly, capsid proteins encoded by ORF2 self-assemble into homodimers through noncovalent interactions at the cytoplasmic membrane [35]. In our study, Western blot analysis showed that HEp148 expressed by HAV-HEp148 in both Huh-7 and Vero cells (data not show) formed dimers. However, we did not detect monomers at 15 kDa with and without heat treatment. The possible reason may be that the HEV antigen is a conformation epitope, and that this homodimeric epitope dissociated into monomers upon denaturation. Studies have shown that the hydrophobic region at position aa 597–602 of the capsid (ORF2) protein is a core region of dimerization, and is directly engaged in the formation of important conformational neutralizing epitopes [19,20]. These homodimer epitopes did not react with neutralizing McAbs after denaturation into monomers [21]. Therefore, dimerization is important for an effective presentation of HEV neutralization epitopes. Our results show that HEp148 dimers formed conformational neutralizing epitopes. Although mice are usually considered not to be suitable for studying HAV infection, the recently published observation that free HAV virions could reach the liver in seronegative and even in slightly seropositive mice [36], encouraged us to use these small animals for the investigation of the immune response to the recombinant HAV-HEp148 virus in vivo. The test group was immunized with HAV-HEp148 virus at passage 3. The HEp148 gene was stably expressed for at least 5 passages in our study. Based on a previous study that indicated the stability, up to the 25th passage, of a 396 bp fragment inserted into the 2A/2B junction of the HAV vector [26], we expected at least a similar stability of the HEp148 gene within the HAV-HEp148 virus.

After inoculation with the HAV-HEp148 virus, the anti-HAV and HEV IgG titers in the HAV-HEp148 group increased significantly from week 4, suggesting that the HAV-HEp148 virus successfully induced satisfactory immune responses against HAV and HEV. The serum HAV- and HEV-specific IgG effective dose in the HAV-HEp148 group was not significantly different from those of the HA or and HE vaccine groups. These results demonstrated that the HEV antigen did not adversely affect the immunogenicity of the live attenuated HA vaccine. A similar result was observed by Dong et al. [22]. Furthermore, the results suggested that immunization with only the recombinant HAV-HEp148 virus could achieve the same effects as with a live attenuated HA vaccine and subunit HE vaccine, after the primary vaccination. It is reported that IgG antibody titer (174.5) is probably protective against further infection with HEV [37]. In our results, recombinant HAV-HEp148 induced higher anti-HEV titer (1:452.55), which may be protective against HEV infection. 

After primary vaccination, the HAV-HEp148 virus induced specific antibodies against the HAV vector in mice. When boosted with the same recombinant virus, pre-existing immunity against HAV may decrease the expression of the HEp148 antigen and reduce vaccine efficacy. However, in our study, after boost vaccination, HAV-HEp148 still induced similar HEV antibody titers, as did the HE vaccine. This result may benefit from some important advantages of viral vectors, such as high-level production of transgenic protein, induction of robust immune responses [38], and adjuvant effects that enhance the immune response against the transgenic protein [39]. Furthermore, IgA-mediated transport of HAV to the liver occurred in the presence of immunity of HAV-seropositive mice [36]. Our boost result also shows that recombinant HAV avoided pre-existing antivector immunity, and induced robust immune responses against the encoded transgenes. This characteristic could be used to develop vaccine/drug delivery vectors targeting liver. In addition, further studies are in progress to test different vaccination regimens, such as an HE vaccine boost. 

Because of some limitations, we did not analyze the induced neutralizing antibody responses against both viruses. However, some studies have reported that live attenuated HAV vaccination can elicit strong protective immune responses [40,41,42]. Moreover, HAV-HEp148 expressed HEp148, which is a vaccine-related HEV neutralization antigenic epitope, and can induce specific HEV neutralizing antibodies in mice [17,43]. Although not directly tested, we hope HAV-HEp148 also elicits specific HAV and HEV neutralizing antibodies, and we will attempt to detect the neutralizing antibodies in future work.

In summary, this study is the first research to express HEV antigen in an active HAV vector. Our results demonstrated a novel HAV-vectored combination vaccine against HAV and HEV (HAV-HEp148). The HAV-HEp148 virus expressed the HEV neutralization epitope HEp148 located at aa 459–606 of the HEV capsid protein, which was able to form a homodimer. Immunization with the HAV-HEp148 virus induced strong antibodies against HAV and HEV in mice. The recombinant HAV also circumvented pre-existing immunity as vaccine delivery vector. Future work is required to detect the induced neutralizing antibody responses against both viruses. It is also necessary to test different vaccination regimens, such as an HE vaccine boost. Such a new approach may lead to the development of combined vaccines and drug transporting system targeting liver.
